# Synchronization and cyclicality of social spending in economic crises

**DOI:** 10.1007/s10663-022-09545-w

**Published:** 2022-08-06

**Authors:** Luis Ayala-Cañón, María Jesús Delgado-Rodríguez, Sonia De Lucas-Santos

**Affiliations:** 1 Facultad de Derecho, UNED, OC/Obispo Trejo 2, 28040 Madrid, Spain; 2 Facultad de Ciencias Jurídicas Y Sociales, Universidad Rey Juan Carlos, Paseo de los Artilleros, s/n, 28032 Madrid, Spain; 3 Facultad de Ciencias Económicas Y Empresariales, Universidad Autónoma de Madrid, C/ Francisco Tomás Y Valiente, 2528049 Madrid, Spain

**Keywords:** Social spending, Factor models, Business cycle, Fiscal policy, C38, E62, F44

## Abstract

This paper expands the analysis of the cyclical characteristics of social spending by providing information on its joint behaviour across OECD countries. With this aim we propose the use of dynamic factor analysis and recursive models to estimate synchronization and cyclicality of social policies within a broad perspective. By considering the synchronization of social spending it is possible to assess the short-run characteristics of the joint response to changes in the economic cycle. We find that synchronization of social spending was only possible for advanced economies, achieving the highest countercyclical stabilization effect during the Global Financial Crisis. Emerging market economies are not able to join the synchronized response, maintaining independent and, in most cases, procyclical stances in the behaviour of their social policies.

**Supplementary Information:**

The online version contains supplementary material available at 10.1007/s10663-022-09545-w.

## Introduction

COVID-19 has brought to the fore the interest in social spending since it is a key policy lever for alleviating the impact of economic crises, preserving economic capacity and protecting the most vulnerable (IMF 2020). International organizations have claimed further and coordinated action to deal with the crisis and be prepared for future threats. Multilateral collaboration and coordination are considered vital to increase the effectiveness of countries´ responses to recovery and strengthen the economy. In this respect, the internationally coordinated G20 Action Plan to deal with COVID-19 is expected to have large stabilizations effects (IMF 2021).

From a Keynesian perspective, there is a clear view that public expenditure should act as a stabilizing force and move in a countercyclical direction. This can be, even more interesting, in the specific case of social spending. If social spending is pro-cyclical, the likelihood of cutbacks in expenditure and less social protection when unemployment and inequality increase is higher. In contrast, when social spending is countercyclical, social benefits comprising discretionary stimuli and the operation of automatic stabilizers contribute to offsetting the fall in household income. This would be a desirable feature from a fiscal stabilization perspective.

Fiscal policies tend to be countercyclical in advanced economies, which helps to smooth out output fluctuations and, thus, yield social gains (Bashar et al. 2017; Jalles 2020a). The effect of cyclical behaviour of fiscal policy among developing and emerging market countries is a much more disputed issue. Several studies have documented that fiscal policy tends to be procyclical or, at best, acyclical in developing and emerging market countries (Bergman and Hutchinson 2020; Gootjes and de Haan 2022). The differences in cyclical behaviour between countries can be explained through different channels. Limited access to external borrowing, fiscal constraints, political distortions, and weak institutions are among the reasons that shape the pattern of cyclicality in these economies (Lane 2003; Ahmad et al. 2021; Dzhambova 2021). Despite the widespread evidence, these studies have focused mainly on measuring the national response of fiscal policies to the business cycle, regardless of the possibility that countries can provide joint stimuli by cooperating with their policies. It is important, thereby, to explore the stabilizing effect of the fiscal stimuli and to identify the countries that participate in those actions to understand the importance of coordination to provide joint capacity for countercyclical policy during economic recessions.

The Global Financial Crisis (GFC) in 2008/09 and the COVID-19 pandemic have shown how interconnected the world is and how quickly downturns in a country can be spread internationally. This can imply both limits on the policymakers´ ability to undertake stabilization policies at the national level, and the need for more international policy cooperation to provide joint public spending stimuli. The latter can be achieved by synchronizing the social spending growth rates over time. The better coordination among the fiscal policy, the more synchronized social policies are likely to lead to significant stabilization effects. In this context, cooperation across countries can increase the stabilization effect of the countercyclical response, especially when a large shock is common across countries. What happened during the GFC offers the possibility to study a coordination experience in which an unprecedented joint fiscal expansion was implemented. Were the different national policies synchronized? Did this possible synchronization make it possible to intensify the stabilizing effects of countercyclical social policies?

This paper tries to answer these questions by measuring the synchronization of social spending and its cyclicality over more than two decades (1980–2013), with a particular focus on how social spending reacted to the GFC. In addition, not all countries had the same possibilities or made the same efforts to provide the requested response to the changes in the economic cycle and contribute to the joint stimuli. A natural question is which countries did so, and to what extent. Providing evidence on these issues contributes to improving the surveillance of social spending and guiding policymakers with a more accurate assessment of cyclical developments.

This paper is motivated by these concerns and offers a framework to investigate the synchronized behaviour and cyclicality of social spending and to provide new evidence on its short-run dynamics. With this aim, the paper revisits the issue of the cyclical characteristics of social policies in a sample of OECD countries, extending the empirical literature in several important ways. First, it provides a framework to test the existence of a *synchronized* social response to the international business cycle. This goal is addressed by estimating a dynamic factor model to assess whether fluctuations in social spending are synchronized. The novelty of this multivariate approach lies in its ability to identify the countries that share a global behaviour and to analyze its trajectory. In this way, it is possible to confirm the existence of a *synchronized* pattern in social spending without imposing the same behaviour on all the countries of a region or on the whole set of countries studied, as many studies usually assume (Calderón et al. 2017; Arze del Granado et al. 2013). Second, we provide an analysis of their cyclical characteristics in terms of duration, amplitude and intensity which helps us to assess its evolution and check whether the cyclical properties of social spending vary along the business cycle and across countries. Third, we consider an alternative measure of cyclicality by implementing recursive models. This method makes it possible to study how social spending responses to the business cycle have evolved across countries without the need to split the sample into sub-periods, something that has frequently been done in previous studies (Bashar et al. 2017; Carneiro and Garrido 2015). Another advantage of this recursive method is that it overcomes the likelihood of a lagged policy response to the shocks. Additionally, we confirm the stability of the parameters obtained in these models by applying the robustness check proposed by Cendejas et al. (2011) to verify the non-existence of structural breaks.

Despite the relevance of all these issues, to the best of our knowledge the analysis of the short-run dynamics of social spending with a national and global perspective has not received attention in empirical work. By integrating the evidence of synchronization and cyclicality, a notable contribution of our paper, therefore, is identifying countries that share social spending fluctuations and then have the possibility to coordinate the response to widespread shocks. We find clear differences between advanced economies and emerging markets. We observe that only the major advanced economies (US, Canada, UK, Belgium, Germany, France and Sweden) were able to reinforce their national responses by synchronizing their countercyclical growth rates of social spending over time. There is another group of advanced countries (mostly EU countries) that made the effort to participate in the countercyclical synchronized response, although they show other national cyclical stances. In contrast, emerging market economies (such as Chile, Mexico and Turkey) had *independent* paths in their social spending growth rates and show mostly procyclical stances in their national social spending policies.

Our results also show that the stabilization capacity of the joint social spending is stable along the period, varying only in response to the international calls. During the GFC, the advanced countries introduced favourable changes in the cyclicality of their social spending and increases in its degree of countercyclicality to provide the fiscal stimulus. Results for emerging countries reaffirm their difficulties to shift towards more favorable policy stances. Therefore, while international organizations endorse the adoption of expansionary countercyclical social policies to mitigate vulnerability during crises, the ability to react to these calls differs between economies.

The remainder of the paper is organized as follows. Section 2 is a brief overview of the literature. Section 3 presents the data and describes the empirical strategy for documenting and assessing the cyclical behaviour of social spending. Section 4 discusses the empirical results and the robustness checks to validate them. Section 5 concludes.

## Literature review

The positive stabilization effects of countercyclical fiscal policies have been widely featured in the literature, but not for every type of economy. Fiscal policies can stimulate the economy in recession periods and ensure sustainable and balanced growth during expansion periods. However, empirical evidence reveals the limitations that developing and emerging market economies show to implement this type of policy. Several economic and institutional factors are likely contributors to fiscal cyclicality. The most reported in the literature are those related to weak political institutions, incomplete markets and borrowing constraints (Aizenman et al. 2019; Calderón and Nguyen 2016). Balanced budget requirements have led some of these countries to engage in substantial procyclicality in government spending, increasing the severity and duration of the crises, with adverse effects on social indicators (Veg and Vuletin 2014) and economic growth (Brueckner and Carneiro 2017).

Understanding the ability to implement countercyclical government spending has aroused interest in identifying movements away from procyclicality in developing and emerging market countries (Frankel et al. 2013; Carneiro and Garrido 2015; Calderón et al. 2017). For some authors, the effectiveness of increasing government spending to boost the economy during recessions is questionable in the case of these countries. There are potential dangers in increasing government spending in countries whose level of debt might be perceived as unsustainable. Fiscal retrenchment under sovereign risk can be an option to curtail the risk of macroeconomic instability (Corsetti et al. 2013; Bianchi et al. 2021).

The literature on the behaviour of fiscal policy over the business cycle has mainly focused on aggregate government spending, but a growing number of papers are focusing on social spending and its ability to smooth output shocks and to promote stabilization. Furceri (2010) and Furceri and Zdzienicka (2012) showed that social spending on old age, health and unemployment contributes the most to smoothing the effects of macroeconomic shocks. Jalles (2020b) used time-varying measures of the cyclicality of social spending for a sample of 26 advanced countries between 1982 and 2012. He found that health and education spending is generally acyclical, pensions are procyclical, and social protection and welfare spending countercyclical. His findings suggest that the degree of countercyclicality in government´s social spending affects the volatility of output.

Studies comparing the cyclical nature of social policies between regions or groups of countries are also frequent. In these cases, cyclicality is analyzed with panel data models, which offer a summary measure but do not identify the individual behaviour of each country, imposing then the same behaviour on all the countries in the sample. Doytch et al. (2012) studied 200 countries between 1980 and 2008 focusing on health and education and using the fixed effects method. Their results indicate that in middle-income countries education spending was acyclical and health spending procyclical, with the opposite pattern in low-income countries. Alfonso and Jalles (2013) focused on education, health and social security spending using two-step robust system-GMM for a sample of developed and emerging market and developing countries for 1970–2008, finding mostly acyclical behaviour but countercyclical in social security spending, particularly in OECD countries. Arze del Granado et al. (2013) used data on health and education in 145 countries for 1987–2007 and system-GMM estimations, finding that spending on education and health was procyclical in developing countries and acyclical in developed countries. Ahuja and Murthy (2017) used panel estimates with fixed effects for a sample of 19 Asian countries between 1980 and 2012, finding that Asian countries were able to escape the procyclical trap during the 2008 economic crisis. In these countries, countercyclicality was constraint by the high accumulated level of public debt.

This interest has also extended to automatic government spending. The results of Galeano et al. (2021) plotting the cyclical components of GDP and primary spending for 131 countries during 1980–2018 show differences across countries and that the nature of unemployment insurance and social security spending are crucial determinants of government spending cyclicality. D’Addio (2015) used data on social security benefits to study the cyclicality of social spending for a panel of 20 OECD countries between 1982 and 2011 employing one-step robust system-GMM, finding that its countercyclical behaviour is stronger in recessions than expansions**.**

The aforementioned works focus on the national response to the business cycle. One way to strengthen the positive effects of stabilization policies is through coordination. The literature focusing on the benefits of coordination shows that benefits from fiscal stimuli are higher when these policies are coordinated (Triggs 2018). The analysis of fiscal coordination rests on the evidence of significant cross-border macroeconomic effects of fiscal spillovers. Several studies have focused on EMU countries because of their common currency and the fact that a single monetary policy demands a certain degree of fiscal policy coordination among the member states. Using a global vector autoregression model (GVAR), Belke and Osowski (2019) found that German and French fiscal spillovers were stronger in EMU than in non-EMU countries. Alcidi et al. (2015) found that the rationale of fiscal policy coordination should be especially strong in a monetary union since economic interlinkages between member countries are relatively high. Hebous and Zimmermann (2013) developed a GVAR model to explore the effects of fiscal spillovers to find that there are considerable differences in Euro Area countries’ reactions depending on whether they were internationally coordinated or not. Alloza et al. (2019) used a structural VAR framework for a sample of the four major euro area economies and found positive and relatively large spillovers in the euro area, although with significant heterogeneity across countries.

Studies with a more global scope also find sizeable international spillover effects. This is the case of Corsetti and Müller (2014), who used a VAR model focusing on the US as a base country suggesting that unexpected fiscal expansions have large impacts on economic activity in the UK and the euro area. Auerbach and Gorodnichencko (2013) used regime-switching models and a sample of OECD countries finding evidence that fiscal shocks have a larger impact when the country concerned is in a recession. Fazzari et al. (2015) also found with a structural VAR that multipliers were higher than normal during recessions in the US economy during 1967–2011. Despite the fact that a key finding from empirical studies has been that multipliers are state-dependent, works with nonlinear techniques and relaxed assumptions yield different results. Ramey and Zubairy (2018) questioned that US government spending multipliers were higher during periods of economic slack. Ilzetzki et al. (2013) showed that the size of fiscal multipliers depends critically on key country characteristics, such as the degree of development. In a recent work, Reis Gomes et al. (2020) cast doubt on the ability of the government in emerging economies to stimulate economic recovery in bad times using expansionary government spending policies. Their results suggest that in turbulent times the private sector is unlikely to be stimulated by government spending stimuli.

Notwithstanding the interest for the analysis of the cyclicality of fiscal policies, there are relatively few studies focusing on the possibility of synchronization and linkages in these policies.^1^ Our work is related to this approach and so far, the attempts to address this issue are scarce and have failed to confirm the existence of explicit fiscal coordination. Using an impulse response function, Goujard (2017) found that when fiscal consolidation efforts are synchronized across partner countries, fiscal policies have large spillover effects on output. Gambetti and Gallio (2016) used a time-varying VAR model for the period 1994–2014 to study fiscal policy coordination in Germany, France, Spain and Italy observing a lack of fiscal coordination in terms of co-movement.

## Data and econometric framework

### Data

Our empirical analysis is based on the SOCX Database (version 2018/9) of the OECD (2018).^2^ The SOCX database contains information on annual per capita social spending for 35 countries from 1980 to 2013, of which 25 are advanced economies and 10 are emerging market economies.^3^ The GDP and population data are also drawn from the OECD database. GDP and social spending are at constant PPPs (2010) in US dollars.

Both the per capita social spending (SS) and GDP series are log-transformed and differentiated to obtain their cyclical component.^4^ For a better understanding of their behavior we employed the Harding and Pagan (2002) dating method which makes it possible to obtain the turning points of the cyclical behaviour. In the Appendix we show the details on the cyclical characteristics of these variables for every country in our sample. Countries are divided according to their economic status in advanced (Tables 4, 5) and emerging market economies (Table 6). The advanced economies are also classified into European Union member states (Table 4) and the rest of advanced economies (Table 5). The two main cyclical characteristics are duration and amplitude. With this information it is also possible to obtain intensity (this is a concept that jointly analyzes the amplitude and duration of a phase, $\frac{amplitude}{duration}$, providing an additional interpretation of expansions and recessions).

From the results corresponding to the turning points reported we observe that for most of the countries under study there was only one expansive economic business cycle during the time period studied (with the exceptions of Denmark, Portugal, Iceland, Turkey, and Mexico). In the case of social spending, we find only countries with more than once expansive cycle across the advanced economies: Belgium, Germany, Ireland, Luxembourg, Netherlands, Portugal, Canada, US and New Zealand. According to the amplitude, we find the highest amplitudes in the expansion periods (more than 4) in Netherland, Norway, Korea, Slovak Republic and Chile while the minimum is the one corresponding to Poland (1.31). In the case of recessions, the highest were found in Chile (5.82) and Mexico (4.67). and the minimum in Poland (1.47) and Turkey (1.90).

The duration of expansive business cycles varies substantially from a minimum of 8 years (Mexico) to a maximum of more than 20 years in Australia, Ireland, Norway, and Germany. In the case of social spending the minimum is 7 years (New Zealand) and the maximum is of 22 years (Japan and Greece). Finally, we find that the countries with the highest amplitude in expansion periods are Korea (4.4) and Ireland (4.49), while the minimum is that of Italy (1.93) and Greece (1.94). For the recession periods, we find the highest amplitude in Germany (5.15) and the lowest in Poland, Israel and Slovak Republic (1.98).

When comparing differences in social spending between advanced and emerging economies, an important finding is that expansionary periods are less intense than recession periods in the former. In the case of emerging economies, we find the opposite result: expansions are more intense than contractive phases, a result in line with the evidence for these countries reflecting the difficulties to increase social spending in crisis and increased pressure to increase spending in boom times.

### Econometric framework

To assess the existence of coordination in social spending across OECD countries when the international business cycle changes and to identify the countries that participate in it, we propose to measure the synchronization of social spending through a dynamic unobserved component approach. We model the degree of co-movements in social spending using a dynamic factor model in the tradition of Forni and Reichlin (1998), Forni et al. (2000), and Stock and Watson (2011). The dynamic factor model assumes that a small number of unobserved latent factors, $f_{t}$, generate the observed time series through a stochastically perturbed linear structure (Stock and Watson 2011). Formally, it is assumed that the pattern of observed co-movements of a high-dimensional vector of time-series countries, ${X}_{t}=\Delta \mathrm{ln}S{S}_{i.t}$,^5^ can be represented by a few unobserved latent common dynamic factors. The latent factors follow time series processes, which are commonly taken to be a vector autoregressive (VAR) model. The dynamic factor model can be summarized as:

1 $${X}_{t}=\Lambda {f}_{t}+{e}_{t}$$

2 $${f}_{t}=\psi (L){f}_{t-1}+{\eta }_{t}$$

where there are *N* countries, so $X_{t}$ and $e_{t}$ are *N* × 1; there are *m* dynamic factors, so $f_{t}$ and $\eta_{t}$ are *m* × 1; $\Lambda = (\lambda_{1} ,\lambda_{2} , \ldots ,\lambda_{m} )$ is *N* × *m*; $L$ is the lag operator; and the lag polynomial matrix $\psi (L)$ is *m* × *m*. The *i-th* $\lambda_{i}$ are called factor loadings for the *i-th* countries and measure the level of participation of each country regarding co-movements captured by the common factor or factors. The idiosyncratic disturbances, $e_{t} = (e_{1,t} ,e_{2,t} , \ldots ,e_{N,t} )^{\prime}$, are the specific elements of each series contained in a vector. These elements are serially correlated and slightly cross-sectionally correlated with other variables in the model and mutually uncorrelated at all leads and lags, that is, $Ee_{it} e_{js} = 0$ for all *s* if $i \ne s$. They are assumed to be uncorrelated with the factor innovations at all leads and lags, that is, $Ee_{t} \eta _{{t - k}}^{\prime }  = 0$ for all $k$. As we do here, it is common to reduce the number of parameters by estimating the signal-to-noise ratios ${q}_{i,m}=\frac{{\sigma }_{\eta ,i}^{2}}{{\sigma }_{e,i}^{2}}$ (Harvey and Trimbur 2008).

The standard estimation method is maximizing the likelihood of the corresponding model and estimation accuracy via the Kalman filter after a suitable reparameterization of the model in state-space form.^6^Assuming that all the processes in (1–2) are stationary and not cointegrated, we use the GROCER’s Econometric Toolbox (Dubois and Michaux 2019). The common factor or global pattern obtained represents the synchronized behaviour of social spending and it contains information about the short-run dynamics of social spending in terms of co-movements across OECD countries. The countries that do not take part in the synchronized behaviour are countries with an independent social spending growth pattern.

For a better identification of the synchronized behaviour of social spending, we also employ the dating method for the common factor of Social Spending, $\hat{f}_{SS,t}$, obtained in (1–2) model. This method allows us to examine the turning points of the *global* pattern, which helps us to follow and understand its trajectory by comparing the phases of the cooperative actions with the phases of the international business cycle.

Then, in order to determine the cyclicality of social spending our proposal is based on the generalization of the recursive econometric model developed by Andrews (1993) and generalized by Cendejas et al. (2011). We investigate, first, the cyclicality of the synchronized behaviour of social spending, $\hat{f}_{SS,t}$, with respect to the international business cycle, $\hat{f}_{GDP,t}$,^7^ where a linear relationship can be established between the extracted factors and assuming that the dependence relationship can be affected by instability. The following model is proposed:

3 $${\widehat{f}}_{GDP,t}=\delta (\tau ){\widehat{f}}_{SS,t}+u(\tau )$$

where $\tau = \tau_{0} ,\tau_{0} + 1, \ldots ,\tau_{1}$, is a possible moving break date, where $\tau_{0} = \pi T$ and $\pi$ is a trimming, a minimum percentage excluded at the beginning of the sample. Therefore, the parameter stability $\delta$ is assessed in each segment of the sample, avoiding the possible problem of delays in the adoption of fiscal policies to respond to shocks. This procedure allows us to assess the cyclicality of the synchronized response depending on the results obtained for the recursive parameter $\delta (\tau )$ or recursive correlation and their t-statistics. We test whether countries participate in a synchronized countercyclical response ($\delta (\tau ) < 0$) that intensifies the stabilizing effects of national social policies through the cross-country links of the fluctuations.

We extend the model (3) to also determine cyclicality of national social spending,$\Delta \ln SS_{i,t}$, with the estimation of the model for every country:

4 $$\Delta \mathrm{ln}GD{P}_{i,t}={\beta }_{i}(\tau )\Delta \mathrm{ln}S{S}_{i,t}+{\xi }_{i,t}(\tau )$$

The continuum of robust results obtained for the recursive coefficients $\beta_{i} (\tau )$ and their t-statistics make it possible to differentiate the following patterns of cyclicality across countries:

- Countries with a national countercyclical response: $\beta_{i} (\tau )$ < 0.
- Countries with other different responses:

$\beta_{i} (\tau ) = 0$ indicates acyclical response

$\beta_{i} (\tau ) > 0$ indicates procyclical response

By integrating the results of the two previous analyses, four different groups can be defined (Table 1). Groups 1–2 consist of countries with social spending synchronization but with differences according to the cyclicality of their national policies. Group 1 includes countries that managed to implement a national countercyclical social spending policy. We consider that these countries intensified the stabilizing effects of national social policies through the cross-country links of their fluctuations. Group 2 comprises the countries with other national social spending patterns but sharing synchronization which facilitates cooperative actions. These countries made an effort cooperating in the joint social spending stimuli since their national responses were not countercyclical. Groups 3–4 include countries with independent patterns in their social spending. They do not show cooperation in their social spending in terms of co-movements. In Group 3 there are countries with a national countercyclical response. Group 4 includes countries with other different stances.

**Table 1:** Synchronization and cyclicality of social spending responses

		Cyclicality of social spending response	
		(a) Countercyclical $\beta_{i} (\tau ) < 0$	(b) Other stances: $\beta_{i} (\tau ) \ge 0$
Synchronization of social spending response	Synchronized $\delta_{i} (\tau ) < 0$	Strengthened national response (1)	Coordinate response (2)
	Independent	Countercyclical response (3)	Other responses (4)

## Empirical analysis

### Synchronization of social spending

We apply our empirical strategy to capture the synchronized behaviour of social spending across OECD countries over the period 1980–2013. The results of the dynamic factor model in (1–2) are shown in Table 2. The AR idiosyncratic parameters and noise ratio confirm the suitability and dynamics of the model. The non-stationarity of AR parameters in the dynamic factor, $\hat{f}_{SS,t}$, confirms the permanent effect of synchronization in social spending policies. The significance of the factor loadings indicates which countries have a social spending that is co-moving and which do not. The results confirm that all factor loadings are significant and statistically similar only for some of the advanced economies: the US, Canada, UK, Japan, and an important group of EU Member States. On the other side, Denmark, Greece, Finland, Switzerland, Turkey, Australia, and New Zealand follow independent patterns. These countries are then excluded from the model, and therefore they do not appear in Table 2.^8^

**Table 2:** Results of models (1)-(2). Sample period: 1981–2013

Synchronized social spending pattern			
*f*_*t*_ = 1.07(4.91***)* f*_*t* − 1_ − 0.48(− 2.71***) * f*_*t* − 2_ +* η*_*t*_ − 0.92 (− 1.99**) * η*_*t* − 1_			
Countries	Factor loading	AR idiosyncratic parameters	Noise ratios
*European Union*			
Belgium	0.52 (2.4)***	− 0.17 (− 0.95)	0.75 (3.95)***
France	0.62 (2.51)***	0.25 (1.41)	0.78 (3.92)***
Germany	0.48 (2.22)***	− 0.17 (-0.98)	0.78 (3.97)***
Ireland	0.70 (2.98)***	0.01 (0.05)	0.66 (3.84)***
Italy	0.41 (1.81)*	0.32 (1.91)*	0.79 (4.00)***
Luxembourg	0.49 (2.16)**	0.26 (1.51)	0.72 (3.97)***
Netherlands	0.57 (2.44)***	0.23 (1.33)	0.73 (3.93)***
Portugal	0.68 (2.96)***	0.39 (2.34)***	0.58 (3.82)***
Spain	0.84 (3.57)**	0.37 (2.06)**	0.44 (3.57)***
Sweden	0.61 (2.61)***	0.37 (2.22)***	0.68 (3.9)***
*Major advanced economies*			
UK	0.64 (2.96)***	0.47 (2.97)***	0.52 (3.81)***
US	1.07 (4.26)***	0.16 (0.61)	0.2 (2.41)***
Japan	0.57 (2.45)***	0.28 (1.63)	0.71 (3.93)***
Canada	0.55 (2.4)***	0.22 (1.25)	0.7 (3.94)***

In () t-statistics, *significant parameter at 90%, **at 95% and ***99%

**Fig. 1:**
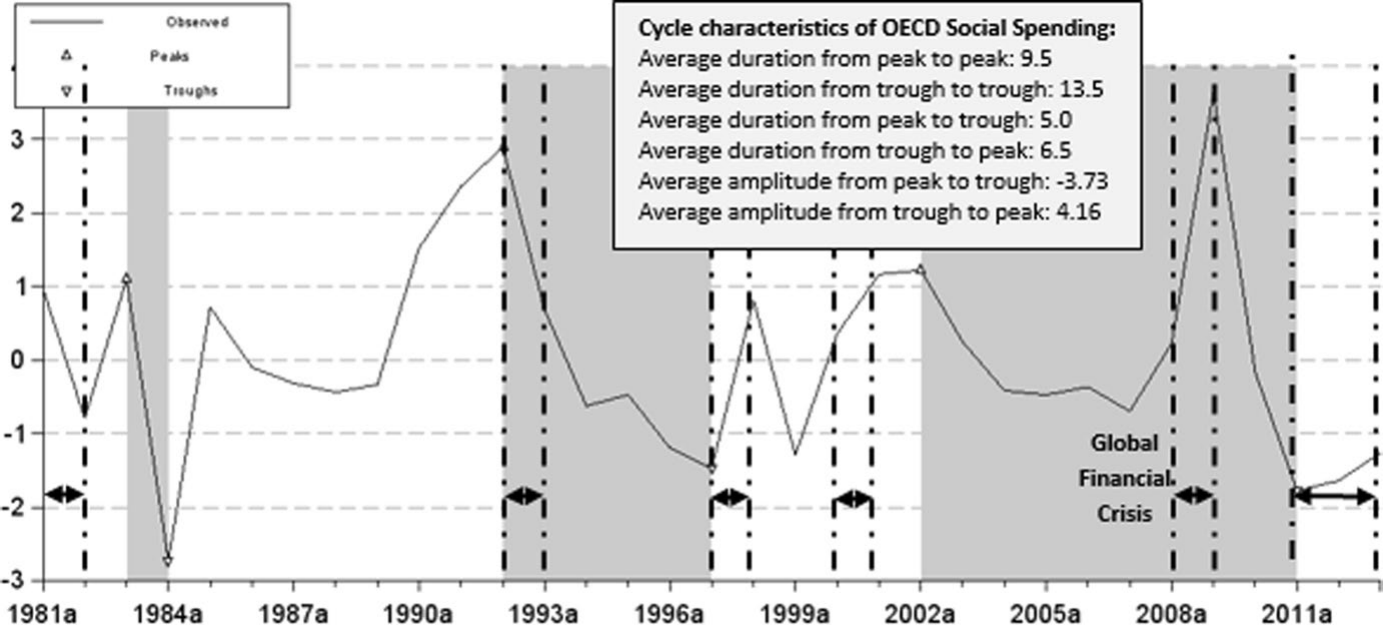
*Synchronized* Social Spending pattern. *Note* Shaded areas correspond to the recession phases in the international social spending pattern, and dashed lines correspond to the GDP recession phases according to the definition in IMF (2019). ^910^ Source: Own calculations in Grocer

The existence of synchronization in the group of advanced economies makes it possible to trace its trajectory during the last decades. Figure 1 shows the evolution of this *synchronized* behaviour over the phases of the business cycle beginning in 1981 and ending in 2013, and its cyclical characteristics. They can be analyzed following the FMI dating methodology and considering social spending dating results. The analysis of the changes in this joint synchronized behaviour confirms that the most successful cooperative action stimulus is related to the call for an international cooperative response in 2008. The G-20 coordinated that action with the objective that the fiscal stimulus resulted in positive spillovers between countries (G-20, 2009).

The analysis of the cyclical characteristics of the synchronized pattern in social spending shows that the average duration of expansions (6.5 years) is higher than that of recessions (5 years), with an average amplitude of expansions (4.16) also greater than that of contractions (− 3.73). This difference implies a greater average intensity of spending in contractions (0.75) than in expansions (0.64). This behavior has been accentuated since the recession of 1992. It is also noteworthy to mention that when comparing the phases of the synchronized social spending with the phases of the business cycle, also shown in Fig. 1, the longest contractions in social spending took place in the period 2002–2011. Even though in response to the 2008 financial crisis advanced countries embraced fiscal stimulus policies, about one year late, most of them adopted austerity measures.

### Cyclicality of social spending

After studying the synchronization of social spending and tracing its evolution, an interesting issue is assessing both the cyclicality of this synchronized behaviour and that corresponding to the 35 national social spending trajectories. With this aim, we estimate recursively the cyclicality over the period under study. The recursive procedure proposed allows us to explore the nature of the social spending responses to the business cycle and whether they vary over time.

**Fig. 2:**
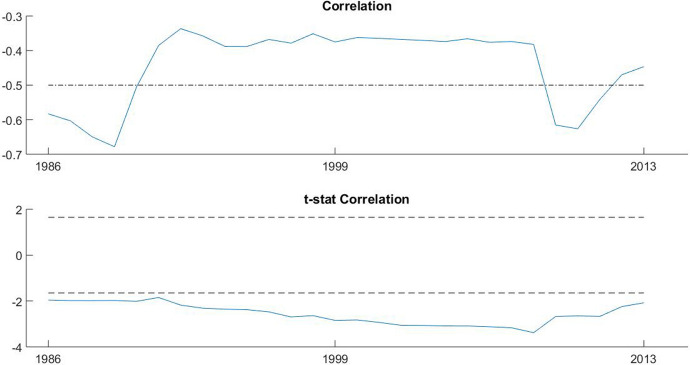
Cyclicality of the *synchronized* Social Spending pattern (model (3)). *Note* Dash-dot lines indicate 0.5 and − 0.5 correlations, and dashed lines indicate 5% significance. Source: Own calculations in Matlab

Figure 2 shows the cyclicality of the synchronized response of the advanced economies to the international business cycle. First, our analysis confirms that the global pattern for these economies was countercyclical during the whole period. The average correlation for most of the period under study is approximately 0.35. We observe a stable correlation during recessions and expansions which shows a similar stabilization capacity of social spending policies. However, we find a marked increase in the correlation during the GFC in 2008, when the countercyclicality of the response increased the most (a correlation of 0.6). These results show that countercyclicality is higher when the cooperative actions are intensified by the international calls and that the stabilization effects of the joint stimulus are higher in slump times, as it is frequently exposed by the literature. These findings also confirm the relevance of the surveillance of international organizations to facilitate joint responses to widespread shocks, reinforcing the stabilizing effects of social spending policies and reducing vulnerability to crises. With the coordinated action, the international organization sent a positive signal to the market, informing how the countries were going to react. However, the degree of countercyclicality decreased strongly when countries reduced their efforts and adopted austerity measures.

**Fig. 3:**
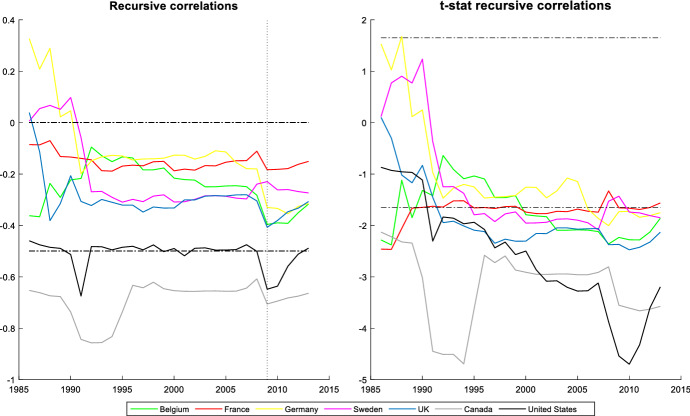
National cyclical response of countries with a *synchronized* pattern (model (4)): Countercyclical stance (at 5% significance and 20% initial trimming). Source: Own calculations in Matlab

**Fig. 4:**
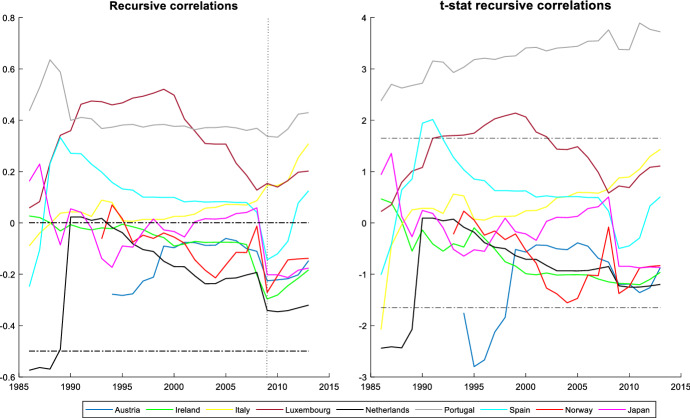
National cyclical response of countries with a synchronized pattern (model (4)): other stances (at 5% significance and 20% initial trimming. Source: Own calculations in Matlab

**Fig. 5:**
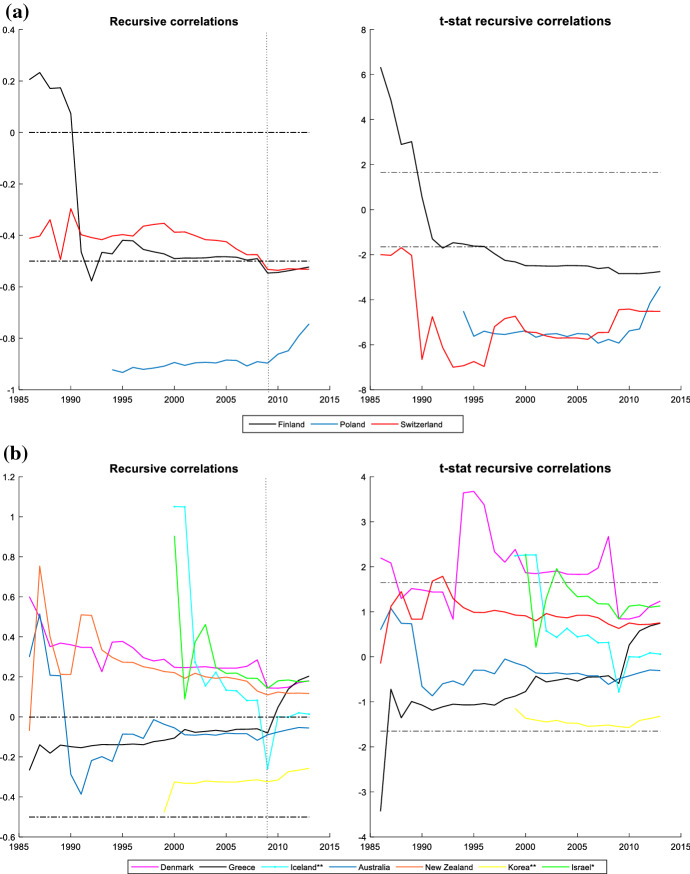
(**a**) National response of countries with an independent pattern (model (4)): countercyclical stance (at 5% significance and 20% initial trimming. (**b**) National cyclical response of countries with an independent pattern (model (4)): other stances (at 5% significance and 20% initial trimming). *Note* *trimming at 30% and **trimming at 40 Source: Own calculations in Matlab

**Fig. 6:**
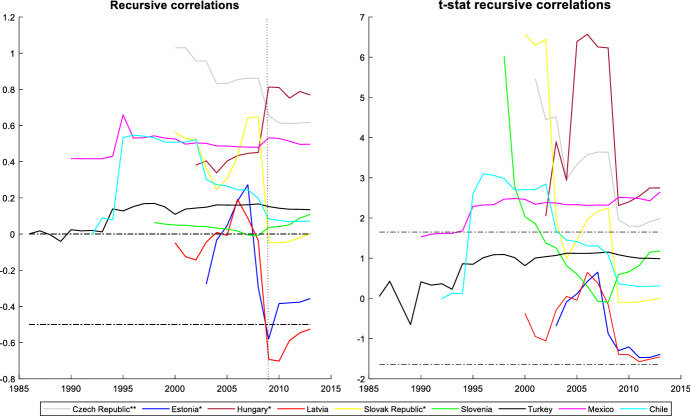
National cyclical response of countries with an independent pattern (model (4)): other stances (at 5% significance and 20% initial trimming. *Note* *trimming at 30% and **trimming at 40 Source: Own calculations in Matlab

It is also interesting to assess the cyclicality of national social spending. It can provide valuable information since it allows us to compare cyclicality across economies. The results are shown by dividing the countries into two groups: those that participate in the *synchronized* behaviour (Figs. 3 and 4) and countries with an independent social spending growth rate (Figs. 5 and 6). The first group is comprised of advanced countries while the second comprises mainly emerging economies, but we also find some advanced economies in it. In both groups we find countries that maintained a national countercyclical behaviour (A), and countries that were either acyclical or, in the worst case, procyclical (B). Thus, national cyclical behaviour does not preclude the possibility to participate in the joint response to external shocks.

In Fig. 3, we show the cyclicality of the *synchronized* countries with a national countercyclical response: Canada, US, UK, Sweden, France, Germany, and Belgium. Among these countries, Canada and US are the economies with a highest countercyclical pattern. These countries show a countercyclical stance during the whole period, with the only exception of Germany and Sweden that showed an acyclical behaviour during the initial years of the period studied. Another important feature of this group is that their degree of countercyclicality is quite stable. We only find increases in its degree of countercyclicality to respond to the GFC although it is later significantly reduced. The strength with which this process occurs varies across countries, being more intense in the US, Canada and UK.

In Fig. 4 we find countries that participate in the coordinated action but have acyclical (Austria, Ireland, Italy, Luxembourg, Netherland, Spain, Norway, and Japan) or procyclical behaviour (Portugal). We do not observe changes in their stances, with the only exceptions of Luxembourg and Spain, which are procyclical during some years in the 1990s, and Austria and Netherland, which are countercyclical for a few years in the mid-1990s. In the case of Portugal, its degree of procyclicality remains high and stable during the years under study, with little changes even during the GFC. European Union rules and the loss of confidence of foreign investors led to extreme austerity in Portugal increasing procyclicality at the end of the period. These are the countries that tried to coordinate their social spending with the global pattern, following the indications of international organizations to increase the social spending to contribute to the fiscal stimulus during the GFC.

Figure 5 includes the countries with a countercyclical response to their national economic cycles but not sharing synchronization with the global pattern: Switzerland, Finland and Poland. Even though all three are countercyclical, they follow different trajectories and degrees of countercyclicality during the years studied. Switzerland is the country with the highest countercyclicality but with some differences in its degree depending on whether the economy is in a recession or expansion stage. In the case of Poland, we also observe a high countercyclical stance along the whole period, although the degree of cyclicality is significantly reduced after the GFC. Poland is one of the few countries that were not directly affected by this crisis, which can explain its reduction of countercyclicality. On the other hand, Finland shows clear changes in its cyclicality. The beginning of the period shows a procyclical stance that changes into acyclical during a brief period and finally is countercyclical since the beginning of the 1990s.

Finally, in Fig. 6B-I we include a very heterogeneous group of advanced and emerging market countries that do not participate in the synchronized response. Some of these countries follow an acyclical stance and others follow a procyclical behaviour. In Fig. 6B-II we present results obtained for the advanced countries. In this group we find Greece, which shows a countercyclical behaviour at the beginning of the period but changes soon to acyclical. Fiscal policy in Greece was highly determined by the EU rules and difficulties with market access. We also find Denmark in this group, showing a marked procyclical stance for its social spending during most of the period. However, the GFC introduced a change in its behaviour becoming acyclical. In the cases of Australia, New Zealand and Korea, we find an acyclical pattern. Australia did not experience a large economic downturn or a financial crisis during the GFC. However, the pace of economic growth did slow significantly. New Zealand and Korea entered a recession after the GFC, but they do not show significant changes in their cyclicality during the GFC. Israel and Iceland showed a high procyclical stance at the beginning of 2000, but rapidly changed to an acyclical behaviour.

Figure 6B-II shows the results obtained for the group of emerging economies. In this group we find emerging European economies (Czech Republic, Estonia, Hungary, Latvia, Slovak Republic and Slovenia). The analysis of these countries is limited by a shorter sample period, but results show that Slovak Republic and Slovenia are procyclical at the beginning of the period and changed to acyclical during the rest of the years studied. Latvia and Estonia are acyclical during the whole period. Ff greater concern are the cases of Hungary and Czech Republic, with procyclical stances during the whole period. In Hungary procyclicality increased during the GFC, being the country with the highest degree of procyclicality in the OECD. In Czech Republic procyclicality reduced significantly for most of the period, but after the GFC this process slowed down and in the last years studied Czech Republic still maintains a high degree of procyclicality.

This group of emerging economies also includes Mexico, Chile and Turkey, countries for which the sample periods are different. Results for these countries show that they are procyclical. In Mexico and Turkey, the degree of procyclicality is stable, failing in reducing its high levels during the GFC. Chile maintained procyclical social spending throughout the period, but with a stage of increased procyclicality until the mid-1990s followed by a reduction but without changing to a more favorable stance. Among these countries, Mexico is the one that reached the highest degree of procyclicality.

These findings are consistent with those of previous studies from the cyclicality literature, which claim that countercyclical behaviours are associated mainly with advanced economies (US, Canada, UK, and France), while a more procyclical stance is found in emerging market countries (Mexico, Chile, Turkey and Hungary). Our results also show that the GFC has introduced favourable changes in the cyclicality of the advanced economies. Either because the countries changed from procyclical to an acyclical stance (Luxembourg and Spain), or from acyclical to countercyclical stance (Finland). In the case of countries with a countercyclical trajectory, they increased their degree during the GFC contributing significantly to the joint fiscal stimulus (UK and US). However, in the case of the emerging countries, their trajectories remain procyclical in most of the cases and the GFC did not improve their results, like in Chile and Mexico. This reaffirms the difficulties of these countries, especially Latin American economies, to shift towards more favorable policy stances.

### Strengthening countercyclical social spending responses

As mentioned above, under the proposed framework we can classify countries according to their results with respect to the short-run dynamics of social spending: cyclicality and synchronization. To do so, we follow the classification presented in Table 1. Table 3 summarizes the results for four groups of countries.

**Table 3:** Synchronization and cyclicality across OECD economies

		Cyclicality of social spending response	
		(a) Countercyclical $\beta_{i} (\tau ) < 0$	(b) Other stances $\beta_{i} (\tau ) \ge 0$
Synchronization of social spending response	Synchronized $\delta_{i} (\tau ) < 0$	Canada US UK Belgium France Germany Sweden (1)	Austria* Ireland Italy Luxembourg Netherlands Norway* Portugal Spain Japan (2)
	Independent	Finland Poland* Switzerland (3)	Denmark Greece Iceland* Estonia* Hungary* Czech Rep.* Slovak Rep* Slovenia Turkey Latvia* Chile* Mexico* Australia New Zealand Korea* Israel* (4)

*Countries with different a subsample period

The first group (1) comprises the countries that strengthened their countercyclical social spending response by synchronizing it with other countries. These countries can be considered naturally coordinated and signed the main agreements as a result of the international calls during the period considered. These countries have the advantage that their national fiscal policies were moving in the same direction to respond to the common shock. The countries that belong to this group are the US, Canada, UK, Germany, Sweden, Belgium, and France, which are the countries that better managed the recovery from the Global Financial Crisis. The countries in this group have a high degree of countercyclicality.

The second group of countries (2) succeeded to share the synchronized behaviour of social spending and benefited from the cooperative actions, although their national responses were mostly acyclical (Austria, Norway, Japan, Spain, Ireland, Italy, The Netherlands, and Luxembourg) or procyclical (the only case is Portugal). The countries in this group had difficulties implementing national countercyclical social spending policies but their social spending was synchronized, showing their commitment to the international objectives of stabilizing demand and supporting social and political stability. The countries in this group made a greater effort to join the fiscal stimulus demanded during the GFC. In the case of the EU Member States, they were encouraged by the fiscal commitments imposed by the Maastricht Treaty and the Stability and Growth Pact. Groups (1) and (2) are made up of high-income countries.

Groups 3 and 4 include countries that did not take part in the joint fiscal stimulus. The group (3) comprises advanced (Switzerland and Finland) and emerging market economies (Poland). In these countries, fiscal policies are determined entirely domestically. They implemented national countercyclical policies, but with *independent* social spending growth rates.

The countries in the last group (4) had more difficulties to implement national countercyclical policies. This group includes advanced (Greece, New Zealand, Israel, Australia) and emerging economies (Estonia, Chile, Mexico, Hungary, Turkey, Latvia, Slovak Republic, Slovenia and the Czech Republic). The lack of synchronization in their social spending policies made it harder to react countercyclically to the GFC and benefit from joint stimuli.

### Robustness checks

We performed different robustness checks to test the validity of our results. First, we confirmed the existence of only one common factor, $\hat{f}_{SS,t}$, by employing the statistical criterion proposed by Bai and Ng (2007). According to these authors, the number of dynamic factors, $p$*,* is $p \le r$, where $r$ is the number of static factors determined by Bai and Ng (2002), where $p = 1$ since $r = 1$ according to the following criteria:

$$ \begin{gathered}   IC_{{pl}} (q) = \log \left( {\det \left( {\sum {} } \right)} \right) + q\frac{{(N + T)}}{{nT}} + \log \left( {\frac{{nT}}{{N + T}}} \right) \hfill \\   IC_{{pl}} (q) = \log \left( {\det \left( {\sum {} } \right)} \right) + q\frac{{(N + T)}}{{nT}} + \log (\min (n,T)) \hfill \\   IC_{{pl}} (q) = \log \left( {\det \left( {\sum {} } \right)} \right) + q\frac{{\log (\min (n,T))}}{{(\min (n,T))}} \hfill \\  \end{gathered}  $$

where $$\sum = variance matrix of residual e_{t}$$.

The next step is to confirm the stability of the parameters and to verify the non-existence of structural breaks. With this aim, we applied the robustness check proposed by Cendejas et al. (2011) to observe changes in the participation of the countries in the synchronized pattern over the period analyzed. For this purpose, we apply the following robustness check:

5 $$\Delta \mathrm{ln}S{S}_{i,t}={\alpha }_{i}(\tau ){f}_{SS,t}+{v}_{i,t}(\tau )$$

From the results for the recursive coefficients $\alpha_{i} (\tau )$ and their t-statistics it is possible to test whether the fluctuations of social spending in every country follow the global pattern over the sample period.

**Fig. 7:**
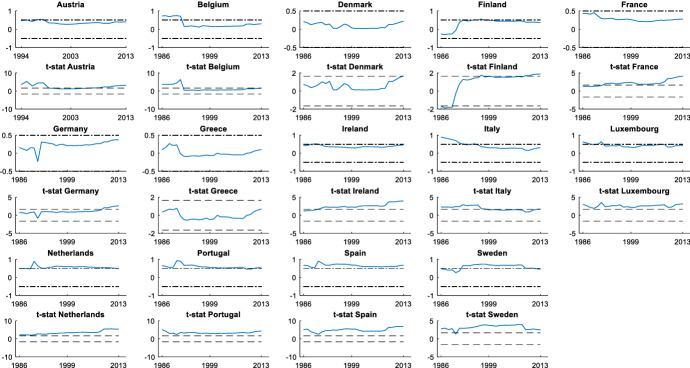
Estimation of recursive parameters (model (5)) in EU Advanced Economies (at 5% significance and 20% initial trimming). *Note* Dash-dot lines indicate 0.5 and − 0.5 correlations, and dashed lines indicate 5% significance. The period studied in this analysis depends on the data availability provided by the OECD Social Expenditure Database (SOCX). Source: Own calculations in Matlab

**Fig. 8:**
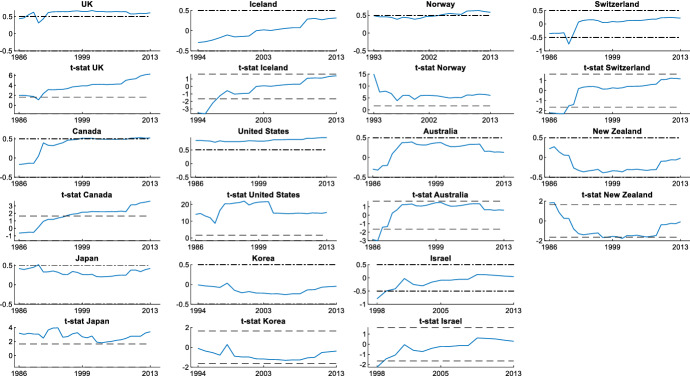
Estimation of recursive parameters (model (5)) in Other Advanced Economies (at 5% significance and 20% initial trimming). *Note* Dash-dot lines indicate 0.5 and − 0.5 correlations, and dashed lines indicate 5% significance. The period studied in this analysis depends on the data availability provided by the OECD Social Expenditure Database (SOCX). Source: Own calculations in Matlab

**Fig. 9:**
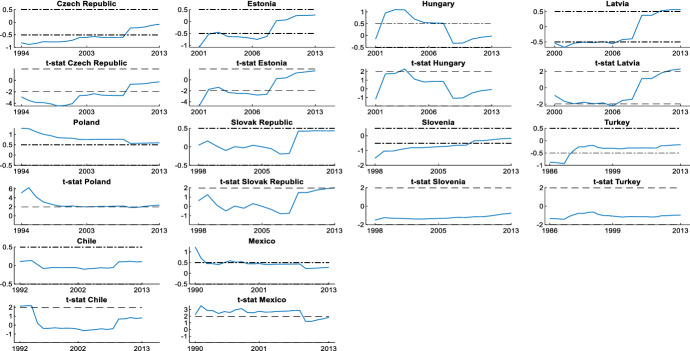
Estimation of recursive parameters (model (5)) in Emerging economies (at 5% significance and 20% initial trimming). *Note* Dash-dot lines indicate 0.5 and − 0.5 correlations, and dashed lines indicate 5% significance. The period studied in this analysis depends on the data availability provided by the OECD Social Expenditure Database (SOCX). Source: Own calculations in Matlab

These results are reported in Figs. 7, 8, 9. Figures 7A and B show the results for the advanced economies and Fig. 9 for the rest of OECD countries. The recursive estimation is useful to confirm the contribution of every country to the *synchronized* social spending behaviour and to verify the existence of ruptures that can affect the model. First, our estimates confirm that Denmark, Greece, Finland, Switzerland, Turkey, Australia and New Zealand are countries that do not share the joint synchronized social spending behaviour. In contrast, the US is the country with the highest contribution to the joint social spending behaviour during the years studied. We also find that a good number of the advanced EU member states make a stable and high contribution to the *synchronized* social spending pattern during the whole period.

This recursive estimation is also useful to extend the analysis to the countries for which the full sample period was not available. The lack of full sample for these countries did not make possible to include them in the estimation of the dynamic factor model. This is the case of Austria, Iceland, Norway, Mexico, Chile, Korea, Poland and Israel. In this way, we can obtain information on the likelihood that these economies also participated in the global pattern during the period analysed. The results show that Austria and Norway are the only countries that participated in the international social spending pattern during their entire period studied. For this reason, we include these countries in the group that shares the synchronized response in Table 3.

## Conclusion

Social spending policies have become a fundamental countercyclical tool for stimulating sustained and equitable recovery from economic crises. The synchronization of these polices across countries can increase their stabilizing effects and contribute to provide joint social spending stimuli. The surveillance of international organizations can be essential to boost a synchronized advance towards broad-based countercyclical national policies that would make them even more effective, as it happened during the GFC and is also expected to happen to mitigate the economic and social impact of the COVID-19 pandemic.

So far, there is much research on the implications of fiscal stimuli for countries, but there is a lack of studies that focus on the stabilization effects of the coordinated actions and the identification of the countries involved. This paper has tried to fill the gap by testing the existence of a synchronized social spending response to the business cycle and analysing its cyclicality. With this aim, we have proposed a framework based on two methodologies. First, for the assessment of the synchronized behaviour it can be used a dynamic factor model that allows to confirm the existence of a global pattern in social spending and the countries sharing it. Second, to study the cyclicality of social spending recursive correlation models have been estimated. This combination of methodological approaches can contribute to broadening the assessment of public policies, providing deeper information on the extent and cyclical characteristics of cooperative actions.

Our empirical analysis leads to different results. First, we find that there was a synchronization in the fluctuations of social spending across most of the advanced countries over the period studied that facilitated an unprecedented expansive joint response during the 2008/09 crisis. The analysis has shown that the synchronization was countercyclical during the whole period and have also confirmed that countercyclicality is higher when it responds to international calls for cooperative coordination to face widespread shocks. Therefore, international surveillance made it possible to undertake more stimuli than countries would have been able to achieve otherwise. This implies that countries can better stimulate the economy by making coordinated actions than taking actions on their own.

Second, not all countries participate similarly in the joint actions. We find that the major advanced economies share a substantial synchronization in their social spending trajectories (US, Canada, UK, Belgium, Germany, France and Sweden). These countries have a prominent role in international organizations, and they also follow countercyclical stances in their national social spending policies. Participating in the joint response, we find a second group of countries that comprises EU Member States and Japan though their national policies followed other stances. This second group is more interesting in terms of coordination, and it contains the countries that make greater efforts to join the G-20 stimulus commitment.

The analysis of the cyclical characteristics of the synchronized social spending behaviour shows that the intensity of contractions in social spending are greater than that of expansions. This behaviour was fostered by the austerity measures put into action during the Global Financial Crisis which contributed to the worsening of the situation in 2011. This result needs to be considered for future crises, as early and strong contractions in social spending can reduce the possibilities of mitigating vulnerability and supporting recovery. It is essential that international and national organizations maintain fiscal stimuli as required.

Finally, we find a group of OECD economies that follow independent paths in their social spending polices. They are mainly emerging market economies and follow procyclical national social policies. The analysis performed showed that these countries had more difficulties to move towards countercyclical responses and benefit from joint social spending stimulus. These countries need to engage in larger efforts to move towards a more countercyclical stance.

The incorporation of synchronization into the analysis of the short run-characteristics of fiscal policy also provides valuable information for a better understanding of how these policies behaved across countries during the last decades and especially during the *Global* Financial Crisis. Future research in this area should consider the specific dynamics of each country in the model to delve into the differences in the social spending responses over the business cycle. Another issue deserving future attention in the analysis of the cyclicality of social spending is the study of the differences in how government transfers—cash and in-kind—have been handled over the business cycle. Finally, the difficulties encountered in moving towards more stable countercyclical social spending policies, mainly in emerging economies, make it necessary to explore what macroeconomic, financial, institutional, and political variables determine their cyclicality. In this sense, improvements in the synchronization of social policies and, in general, in the coordination of fiscal policy may be another factor to facilitate movements towards more countercyclical stances, contributing to reinforce the positive international effects of fiscal policies.

### Electronic supplementary material

Below is the link to the electronic supplementary material.

Supplementary file1 (CSV 15 kb)

Supplementary file2 (CSV 11 kb)

Supplementary file3 (XLSX 199 kb)

## Appendix

See Tables 4, 5, 6 and 7.

**Table 4:** Cycle Characteristics of Social Spending and GDP. EU advanced economies

	Social spending							GDP						
	Turning points		Duration			Amplitude		Turning points		Duration			Amplitude	
	Peak	Through	Exp	Cont	Cycle	Exp	Cont	Peak	Through	Exp	Cont	Cycle	Exp	Cont
Austria	1999	2005		6			2.9	1990	1993		3			2.1
	2009	2011	4	2	6	2.0	2.4	1998	2009	5	11	16	2.0	4.1
	Median		4.00	4.00	6.00	1.96	2.64	Median		5.00	7.00	16.00	2.01	3.06
Belgium		1984						1988	1993		5			3.2
	1991	1992	7	1	8	2.44	3.5	1997	2009	4	12	16	2.7	3.7
	2009	2012	17	3	20	3.87	2.3							
	Median		12.00	2.00	14.00	3.16	2.90	Median		4.00	8.50	16.00	2.68	3.41
Denmark								1986	1988		2			2.3
	1994	1997		3			4.6	1994	2002	6	8	14	2.5	2.4
	1999	2012	2	13	15	2.4	2.4	2006	2009	4	3	7	1.7	4.5
	Median		2.00	8.00	15.00	2.45	3.50	Median		5.00	3.00	10.50	2.07	2.36
Finland	1982	1995		13			3.6	1988	1991		3			3.7
	2002	2011	7	9	16	2.7	1.0	1997	2009	6	12	18	4.0	4.8
	Median		7.00	11.00	16.00	2.67	2.32	Median		6.00	7.50	18.00	3.98	4.22
France	1985	1989		4			5.5	1988	1993		5			3.12
	1995	2006	6	11	17	2.6	2.6	1998	2009	5	11	16	2.56	4.06
	2009		3			1.9								
	Median		4.50	7.50	17.00	2.25	4.03	Median		5.00	8.00	16.00	2.56	3.59
Germany	1985	1991		6			3.2		1982					
	1992	2006	1	14	15	5.3	4.5	1990	2009	8	19	27	2.4	5.2
	2009	2011	3	2	5	2.4	2.6	2010		1			5.2	
	Median		2.00	6.00	10.00	3.85	3.19	Median		4.50	19.00	27.00	3.78	5.15
Greece		1988						1988	1993		5			1.5
	2001	2010	13	9	22	3.2	3.3	2003	2011	10	8	18	1.9	3.8
	Median		13.00	9.00	22.00	3.22	3.34	Median		10.00	6.50	18.00	1.94	2.67
Ireland		1989							1983					
	1992	2000	3	8	11	1.5	1.2	1999	2008	16	9	25	2.4	3.6
	2008	2011	8	3	11	2.1	2.9	2015		7			6.6	
	Median		5.50	5.50	11.00	1.84	2.05	Median		11.50	9.00	25.00	4.49	3.59
Italy		1995						1988	1993		5			2.1
	1997	2011	2	14	16	3.3	3.0	2000	2009	7	9	16	1.9	4.2
	Median		2.00	14.00	16.00	3.27	2.96	Median		7.00	7.00	16.00	1.93	3.16
Luxemburgo		1982						1986	1996		10			2.6
	1991	1996	9	5	14	3.5	2.5	1999	2009	3	10	13	1.9	3.8
	2002	2011	6	9	15	2.3	3.6	2014		5			2.8	
	Median		7.50	7.00	14.50	2.92	3.04	Median		4.00	10.00	13.00	2.38	3.24
Nehterlands		1984							1982					
	1990	1994	6	4	10	5.0	5.3	1989	1993	7	4	11	3.0	1.8
	2005	2008	11	3	14	3.3	2.1	1999	2009	6	10	16	2.1	4.8
	Median		8.50	3.50	12.00	4.15	3.68	Median		6.50	7.00	13.50	2.56	3.30
Portugal		1982							1984					
	1990	1994	8	4	12	4.9	3.7	1988	1993	4	5	9	2.9	2.9
	2000	2012	6	12	18	1.8	2.7	1998	2012	5	14	19	1.9	2.4
	Median		7.00	8.00	15.00	3.34	3.24	Median		4.50	9.50	14.00	2.41	2.63
Spain		1984						1987	1993		6			
	1990	1994	6	4	10	4.0	4.7	2000	2009	7	9	16	2.6	2.8
	2009		15			3.4		2015		6			3.5	3.9
	Median		10.50	4.00	10.00	3.70	4.75	Median		6.50	7.50	16.00	3.04	3.35
Sweden	1985	1995		10			4.8	1984	1993		9			3.1
	2003	2007	8	4	12	3.0	2.2	2000	2009	7	9	16	3.3	4.9
								2015		6			4.4	
	Median		8.00	7.00	12.00	3.00	3.49	Median		6.50	9.00	16.00	3.86	4.01

**Table 5:** Cycle Characteristics of Social Spending and GDP. EU advanced economies

	Social spending							GDP						
	Turning points		Duration			Amplitude		Turning Points		Duration			Amplitude	
	Peak	Through	Exp	Cont	Cycle	Exp	Cont	Peak	Through	Exp	Cont	Cycle	Exp	Cont
UK	1983	1988		5			3.4	1988	1991		3			3.2
	1992	1997	4	5	9	4.0	3.2	1997	2009	6	12	18	2.5	4.2
	2001		4			2.2		2014		5			3.3	
	Median		4.00	5.00	9.00	3.10	3.29	Median		5.50	7.50	18.00	2.93	3.68
**Switzerland**	1992	2006		14			3.7	1989	1991		2			2.8
	2009		3			2.5		2006	2012	15	6	21	2.8	1.7
	Median		3.00	14.00	NaN	2.48	3.66	Median		15.00	4.00	21.00	2.78	2.28
Norway	1998	2000		2			3.4		1988					
	2009		9			4.1		1997	2009	9	12	21	2.8	3.9
	Median		9.00	2.00	NaN	4.14	3.38	Median		9.00	12.00	21.00	2.78	3.92
Iceland									1983					
		1992						1987	1992	4	5	9	3.0	3.4
	2002	2010	10	8	18	2.8	4.2	1997	2009	5	12	17	3.1	3.7
								2016		7			3.6	
	Median		10.00	8.00	18.00	2.82	4.20	Median		5.00	8.50	13.00	3.08	3.52
Canada	1982	1987		5			3.4	1984	1991		7			4.0
	1991	1995	4	4	8	2.4	3.7	1999	2009	8	10	18	3.7	4.1
	1998	2011	3	13	16	2.1	1.5	2011		2			3.1	
	Median		3.50	5.00	12.00	2.27	3.36	Median		5.00	8.50	18.00	3.39	4.05
US		1984												
	1991	1997	7	6	13	3.6	2.9	1984	1991		7			3.8
	2002	2005	5	3	8	2.8	2.2	1999	2009	8	10	18	2.5	3.6
	2009	2011	4	2	6	3.5	4.4	2015		6			3.0	
	Median		5.00	3.00	8.00	3.46	2.86	Median		7.00	8.50	18.00	2.71	3.73
Australia	1983	1988		5			2.8							
	1995	2006	7	11	18	4.1	4.0	1987	1990		3			4.0
	2008		2			2.7		1998	2012	8	14	22	3.9	2.7
	Median		4.50	8.00	18.00	3.41	3.43	Median		8.00	8.50	22.00	3.91	3.34
New Zeland		1987												
	1988	1991	1	3	4	3.3	3.9		1991					
	1997	2001	6	4	10	3.5	2.5	1993	2008	2	15	17	4.4	3.1
	2009		8			2.2		2015		7			2.0	
	Median		6.00	3.50	7.00	3.35	3.21	Median		4.50	15.00	17.00	3.22	3.14
Japan		1984						1988	1998		10			3.3
	1995	2006	11	11	22	3.0	3.6	2004	2009	6	5	11	1.5	3.4
	2009		3			3.5		2010		1			4.2	
	Median		7.00	11.00	22.00	3.25	3.64	Median		3.50	7.50	11.00	2.89	3.34
Korea	1999	2000		1			5.1	1983	1998		15			4.7
	2006	2011	6	5	11	4.0	1.8	1999	2009	1	10	11	4.4	2.7
	Median		6.00	3.00	11.00	4.01	3.47	Median		1.00	12.50	11.00	4.44	3.68
Israel		2004							2001					
	2010		6			2.7		2007	2012	6	5	11	3.3	1.9
	Median		6.00	NaN	NaN	2.73	NaN	Median		6.00	5.00	11.00	3.26	1.93

**Table 6:** Cycle Characteristics of social spending and GDP. Emerging economies

	Social spending							GDP						
	Turning points		Duration			Amplitude		Turning points		Duration			Amplitude	
	Peak	Through	Exp	Cont	Cycle	Exp	Cont	Peak	Through	Exp	Cont	Cycle	Exp	Cont
Cczech Republic									1997					
	1995	2012		17			2.8	2006	2009	9	3	12	1.8	3.1
								2015		6			2.7	
	Median		NaN	17.00	NaN	NaN	2.76	Median		7.50	3.00	12.00	2.26	3.09
Estonia									1999					
	2008	2010		2			3.5	2006	2009	7	3	10	1.8	4.6
	Median		NaN	2.00	NaN	NaN	3.46	Median		7.00	3.00	10.00	1.79	4.57
Hungary									1992					
	2002	2009		7			3.3	2004	2009	12	5	17	2.9	4.3
								2014		5			4.0	
	Median		NaN	7.00	NaN	NaN	3.34	Median		8.50	5.00	17.00	3.48	4.29
Latvia		2000												
	2009	2011	9	2	11	3.3	3.9	2006	2009		3			4.563
	Median		9.00	2.00	11.00	3.27	3.92	Median			3.00			4.56
Poland		2000							2001					
	2001	2011	1	10	11	1.3	1.5	2007	2013	6	6	12	2.1	2.0
	Median		1.00	10.00	11.00	1.31	1.47	Median		6.00	6.00	12.00	2.05	1.98
Slovak Republic	1998	2000		2			2.2	1996	1999		3			2.2
	2009	2011	9	2	11	4.4	3.1	2007	2009	8	2	10	3.4	5.2
	Median		9.00	2.00	11.00	4.40	2.64	Median		6.00	6.00	12.00	2.05	1.98
Slovenia	2010	2012		2			0.2	2006	2009		3			4.563
	Median		NaN	2.00	NaN	NaN	0.23	Median			3.00			4.56
Turkey									1982					
	1990	2000		10			2.9	1987	2001	5	14	19	1.5	3.6
	2003	2010	3	7	10	2.2	0.9	2004	2009	3	5	8	3.8	3.4
								2011		2			3.8	
	Median		3.00	8.50	10.00	2.20	1.90	Median		3.00	9.50	13.50	3.76	3.54
Chile	1995	2003		8			5.8	1992	1999		7			4.1
	2008		5			4.6		2004	2016	5	12	17	2.7	2.1
	Median		5.00	8.00	NaN	4.59	5.82	Median		5.00	9.50	17.00	2.74	3.13
Mexico									1983					
	1989	1995		6			4.7	1994	1995	11	1	12	3.1	3.5
	1998		3			3.4		1997	2001	2	4	6	4.1	2.1
								2006	2009	5	3	8	1.5	3.1
								2010		1			3.3	
	Median		3.00	6.00	NaN	3.40	4.67	Median		3.50	3.00	8.00	3.17	3.11

**Table 7:** Preliminary estimation of model (1)-(2) with the full sample. Period: 1981–2013

International social spending pattern			
*f*_*t*_ = 0.99(3.36***)* f*_*t* − 1_ − 0.48(− 2.76***) * f*_*t* − 2_ +* η*_*t*_ − 0.72 (− 1.99**) * η*_*t* − 1_			
Countries	Loading factor	AR parameters	Residual variance
*European Union*			
Belgium	0.68 (2.4)***	− 0.17 (− 0.96)	0.77 (3.96)***
Denmark	0.4 (1.25)	0.15 (0.85)	0.89 (4.04)***
Finland	0.46 (1.6)	0.4 (2.49)***	0.69 (4.02)***
France	0.91 (2.88)***	0.21 (1.15)	0.75 (3.9)***
Germany	0.72 (2.6)***	− 0.21 (− 1.17)	0.75 (3.94)***
Greece	0.27 (0.89)	0.39 (2.44)***	0.81 (4.05)***
Ireland	1.02 (3.46)***	0.01 (0.04)	0.64 (3.81)***
Italy	0.63 (2.05)**	0.31 (1.79)*	0.77 (3.99)***
Luxembourg	0.66 (2.2)**	0.27 (1.53)	0.73 (3.97)***
Netherlands	0.71 (2.26)***	0.24 (1.38)	0.77 (3.97)***
Portugal	0.87 (2.94)***	0.36 (2.14)**	0.63 (3.89)***
Spain	1.1 (3.89)***	0.34 (1.94)**	0.5 (3.7)***
Sweden	0.96 (3.2)***	0.38 (2.26)***	0.63 (3.85)***
*Other advanced economies*			
United_Kingdom	0.99 (3.69)***	0.46 (2.81)***	0.48 (3.74)***
Switzerland	0.31 (0.97)	0.14 (0.79)	0.91 (4.05)***
Canada	0.85 (2.83)***	0.21 (1.17)	0.67 (3.91)***
United_States	1.49 (5.74)***	0.36 (1.55)	0.2 (2.43)***
Australia	0.12 (0.37)	0.06 (0.34)	0.96 (4.06)***
New Zealand	0.09 (0.29)	0.2 (1.13)	0.93 (4.06)***
Japan	0.81 (2.64)***	0.24 (1.38)	0.72 (3.93)***
Turkey	− 0.23 (− 0.8)	− 0.2 (− 1.16)	0.92 (4.05)***

In () t-statistics, *significant parameter at 90%, **at 95% and ***99%

## Footnotes

1 Synchronization has been addressed in numerous studies to examine the dynamics of business cycles (Anagostou et al. 2015; Antonakakis and Tondl 2014).

2 Available online at http://www.oecd.org/social/expenditure.htm.Social Spending includes Old Age, Survivors, Incapacity Related, Health, Family, Active Labour Market Programmes, Unemployment, Housing, and Other Social Policy Areas.

3 The starting and final years depend on the data availability for each country.

4 This is the “growth” definition and the one that is most frequently employed in the empirical literature on business cycles. In this case, a recession is usually defined as a period of at least two consecutive years of negative growth. See Prescott and Kydland (1990) for a discussion of alternative definitions. Originally, one of the most common approaches was the Hodrick Prescott filter (Hodrick and Prescott 1997). However, this filter suffers for several shortcomings (Hamilton 2018).

5 These series are stationary (log-transformed and differentiated) and are assumed to be non-cointegrated. They are non-cointegrated if the common factor follows an invertible process $MA(\infty )$.

6 A detailed description of the Kalman filter can be found in Clark (1989), and Stock and Watson (1991).

7 This analysis requires information on the international business cycle. We estimated it using model (1)-(2), being in this case the dependent variable $Y_{t} = \Delta \ln GDP_{i.t}$ in Eq. (1), and obtaining a common factor for the business cycle $\hat{f}_{GDP,t}$.

8 The results obtained with the full sample are presented in Table 7 of Appendix.
